# Neutrophil/Lymphocyte Ratio and Platelet/Lymphocyte Ratio in Branch Retinal Vein Occlusion

**DOI:** 10.1155/2019/6043612

**Published:** 2019-10-31

**Authors:** Dan-dan Zhu, Xun Liu

**Affiliations:** Department of Ophthalmology, Drum Tower Hospital Affiliated to Nanjing University Medical School, Nanjing, China

## Abstract

*Purpose*. To evaluate the neutrophil/lymphocyte ratio (NLR) and the platelet/lymphocyte ratio(PLR) value in the development of branch retinal vein occlusion (BRVO)patients. Methods. 81 patients with BRVO and 81 age and sex-matched subjects were recruited as the control group. The BRVO diagnosis was confirmed under comprehensive ophthalmologic examinations. NLR and PLR parameters obtained from peripheral blood were recorded. Results. Both the mean NLR and PLR was significantly higher in the BRVO group compared with the control group (*p* < 0.001). In ROC analysis, the AUC for NLR was 0.82, and NLR of >2.48 predicted BRVO with a sensitivity of 58% and specificity of 98%. The AUC for PLR was 0.78, and PLR of >110.2 predicted BRVO with a sensitivity of 72% and specificity of 72%. Conclusion. The current study demonstrated that BRVO patients had increased NLR and PLR levels compared with control subjects. The NLR and PLR may be used as independent predictors for identifying risk for the development of BRVO.

## 1. Introduction

Branch retinal vein occlusions (BRVO) is the second most common category of retinal vascular disease after diabetic retinopathy, which is an important cause of painless visual loss in middle-aged and elderly individuals [1]. With the changes in life style habits and aging, the incidence of BRVO seems to increase rapidly. Epidemiologic studies have described the prevalence of branch retinal vein occlusion (BRVO) ranges between 0.6% and 1.1% in the Caucasian population [2]. In terms of the prevalence of BRVO in the Asian countries, Nepal was 2.74% [3].

The most serious result of BRVO was irreversible visual impairment and blindness, which was hard to be detected as early as possible by actively targeting and screening the risk individuals since timely intervention can reduce vision-threatening sequelae.

Although the pathological mechanism of BRVO has not been fully elucidated, it has been suggested that inflammatory markers may play a crucial role in the formation and development of this disease [4, 5].

There are some reports which estimated that some common, simple, and inexpensive biomarkers such as platelet/lymphocyte ratio (PLR) and neutrophil/lymphocyte ratio (NLR) are associated with systemic inflammation response [6]. Recently, NLR and PLR values have been recognized as the predictor, treatment response, or prognostic indicators for pretreatment in some types of carcinoma [7–9]. Besides, both these prognostic scores have been testified to be involved in thrombotic processes in cardiovascular disease [10, 11].

Notably, systemic diseases such as atherosclerosis, hypertension, diabetes mellitus, and hyperlipidaemia are the major risk factors for BRVO [12]. The underlying mechanisms for BRVO are regarding that thrombosis and inflammatory processes go with the processes of this disease [13, 14].

On this basis, in contrast, few data regard the prognostic role of these inflammatory parameters. In this study, we aimed to explore the possible link between PLR and NLR values in patients with BRVO.

## 2. Methods

A total of 81 patients diagnosed with branch retinal vein occlusion (BRVO group) between June 2018 and April 2019 were reviewed retrospectively in this study. Written informed consent in accordance with the Declaration of Helsinki was obtained from each participant, and the study was approved by the local ethics board. All subjects underwent a comprehensive ophthalmologic evaluation including best-corrected visual acuity (BCVA), slit-lamp biomicroscopy, intraocular pressure (IOP), dilated fundus examination, intraocular pressure measurement, fluorescein angiography (FA), and macular OCT (Heidelberg Spectralis; Heidelberg Engineering, Heidelberg, Germany).

Fundus features of BRVO include dilated and tortuous retinal vein, cotton-wool spots, sectorial superficial retinal hemorrhages with flame shape arising from arteriovenous crossing, and macular edema.

All patients underwent routine physical examination. Exclusion criteria included a history of diabetes mellitus, smoking, renal failure, hepatic disorders, anemia, malignancy, cardiovascular disease, systemic inflammatory disease, glaucoma, and using nonsteroidal anti-inflammatory drugs and anticoagulant medications. The control group consisted of 81 age-matched subjects from the ophthalmology clinic with early cataract.

Blood samples for complete blood counts were drawn from the antecubital vein before 08:00 a.m after a fasting period of 12 hours. Total white blood cells, neutrophils, lymphocytes, and platelets were measured using an automatic blood analyzer (Sysmex KX-21, Japan). The NLR and PLR were calculated by the ratio of the neutrophils and platelets to lymphocytes.

### 2.1. Statistical Analysis

Statistical analysis was done by SSPS statistical software (ver. 17.0; SPSS Inc., Chicago, IL, USA). Data were presented as means ± SD for continuous variables and as percentages for categorical variables. Group comparisons for continuous variables were expressed by using the Mann–Whitney *U* test for the data did not show a Gaussian distribution. The chi-square test was performed for analysis of variance. The Bonferroni correction was applied for post hoc analysis. Multivariate logistic regression analysis was performed to identify the risk factors for BRVO patients.

Receiver operating characteristic (ROC) curve analysis was used to analyze the optimal cutoff value of NLR and PLR for predicting BRVO, demonstrating the maximum sensitivity and specificity. The predictive validities were quantified as areas under the curve (AUC). *p* < 0.05 was considered statistically significant, and the confidence interval (CI) was 95%.

## 3. Results

The study population included 81 patients diagnosed with BRVO (34 women and 47 men with a mean age of 61.35 ± 1.18 years) versus age- and sex-matched control subjects (30 women and 51 men with a mean age of 59.83 ± 1.15 years). The baseline clinical characteristics of the study patients are shown in Table 1. There were no statistical differences in terms of age and gender (*p* > 0.05) between the groups. The presence of hypertension did not differ between the two groups (*p*=0.73). BCVA was lower in BRVO groups in comparison with control subjects (0.52 ± 0.2 vs. 0.29 ± 0.1, *p* < 0.001), but there were no statistically significant differences between the groups with respect to IOP (*p*=0.43).

The laboratory findings of the study patients are presented in Table 2. There were no statistically significant differences between the two groups with respect to hemoglobin level, mean platelet volume level, platelet level, and white blood cells level (*p* > 0.05). Neutrophil levels were significantly higher in BRVO patients compared to the control subjects (4.31 ± 1.4 vs. 2.94 ± 0.65, *p* < 0.001). Lymphocyte levels were significantly lower in BRVO patients compared to the control subjects (1.88 ± 0.49 vs. 2.41 ± 0.54, *p* < 0.001). The mean NLR was significantly higher in the BRVO group compared to the control group (2.52 ± 1.18 vs. 1.30 ± 0.47, *p* < 0.001). Similarly, the mean PLR was significantly higher in the BRVO group compared to the control group (142.5 ± 45.19 vs. 105.9 ± 34.86, *p* < 0.001).

**Figure 1:**
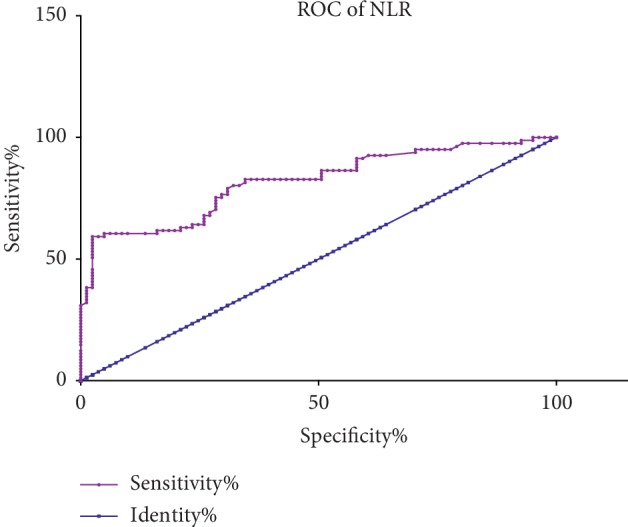
Receiver operating characteristic analysis of NLR in BRVO.

**Figure 2:**
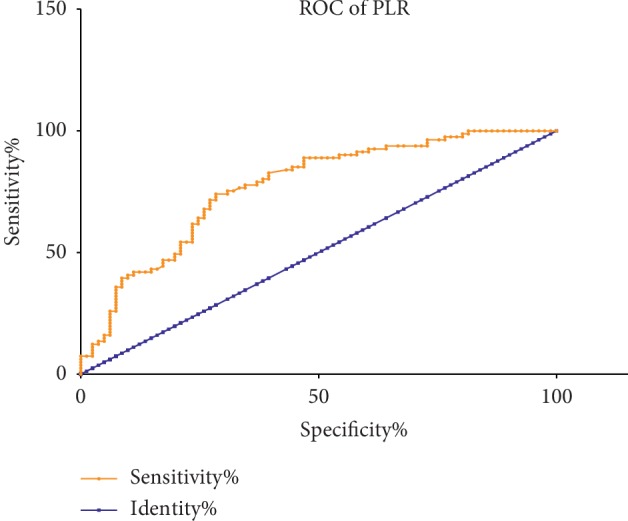
Receiver operating characteristic analysis of PLR in BRVO.

According to the receiver operating characteristic curve (ROC) analysis, the optimal cutoff value of NLR and PLR in the prediction of BRVO was detected. The optimal cutoff value of NLR to predict BRVO was >2.48, with an AUC value of 0.82, 95% confidence interval 0.75–0.88, sensitivity 58%, and specificity 98% (Figure 1). The optimal cutoff value of PLR to predict BRVO was >110.2, with an AUC value of 0.78, 95% confidence interval 0.70–0.84, sensitivity 72%, and specificity 72% (Figure 2).

## 4. Discussion

In our study, we evaluated the importance of NLR and PLR in branch retinal vein occlusion and found that PLR and NLR values were significantly increased in BRVO patients than the control group. NLR and PLR may be the independent predictors of BRVO. These data suggest that the inflammatory and thrombotic process might have a crucial role in the incidence of BRVO.

The white blood cell and its subtypes play a key role in the process of inflammatory response in tumor development and cardiovascular disease [15, 16]. The role of neutrophils during the inflammatory course of acute stress and various tissue damage has been suggested. Khanam et al. [17] demonstrated that during the course and outcome of liver failure, neutrophils were directly implicated in risk of infection and mortality. Recent studies have indicated that neutrophil levels contribute to tumor growth and metastasis by the release of a number of chemokines, including vascular endothelial growth factor (VEGF), IL-8, angiopoietin-1, and matrix metalloproteinase-9 [18, 19]. In line with this notion, the increased levels of vascular endothelial growth factor (VEGF) are responsive to decreased vision for the formation of macular edema due to BRVO. In our study, we found that neutrophil values were significantly higher among the BRVO group than those in the control group, which is consistent with the result by the previous study [20].

And lymphocytes, playing the opposite role with neutrophils, could regulate the inflammatory response and be another strong predictor of cardiovascular risk [21]. Sonmez et al. [22] found that lymphocyte counts decreased because of steroid exposure and increased cell apoptosis, which is also a marker of poor general heath and physiological stress. Our results indicated that lymphocyte values were significantly lower among the BRVO group than those in the control group, which may be supported by studies explaining mechanism of its antiatherosclerotic role [21].

Thus, NLR value, based on the neutrophil and lymphocyte count, is an integrated reflection of different yet complementary immune pathways and more important than either parameter alone. NLR has been proved higher in both stable coronary artery disease and acute coronary syndrome [23]. Besides, Sato et al. [24] suggested that NLR could predict the severity grade independently in patients with acute cholecystitis, which would be important to optimize the treatment and achieve a better prognosis. The predictive superiority of NLR has been associated not only with cardiovascular disease and systemic inflammatory diseases but also with cancer progression and distant metastasis. A retrospective study suggested that pretreatment NLR values, as an independent prognostic factor, were significantly associated with distant metastasis in pancreatic cancer patients [25].

It is believed that the pathological mechanism of retinal vein occlusion may be due to vessel wall degeneration, arteriovenous crossings occlusion, blood hypercoagulability, and thrombogenesis [26]. Epidemiological studies have shown that BRVO was closely related to cascades of inflammation and thrombosis [12, 27]. So, the result of NLR level is clinically important for predicting the development in BRVO patients. Dursun et al. [20] found that NLR was higher in RVO subjects than the control subjects and NLR has 100% specificity for predicting RVO. On the contrary, Kumral et al. [5] got the opposite result. They suggested that there was no difference in NLR values between the BRVO and control group. Interestingly, our data showed that NLR was significantly higher in BRVO patients, and this originated from the presence of lower lymphocyte levels.

Any possible explanation for those conflicting evidence is the small cohort of patients included, which might have influenced the statistical significance. In addition, different systemic examination results should be taken into consideration as those results may have an impact on the patient performance.

Also, it has been known that platelets are critical sources, especially for VEGF, in wound healing process [28]. So, larger and activated platelet release may have an important role in angiogenesis. However, to our knowledge, few studies evaluated the association between platelet/lymphocyte ratio (PLR) level and the progression of BRVO. In our study, we showed that PLR was significantly increased in patients with BRVO. High platelet levels intervene in angiogenesis, tumor growth, and inflammation, which is controlling lymphatic vessel development and produce relative thrombocytosis [29, 30]. Whereas, the present study showed no difference between the two groups in platelet values. The mechanism underlying the relation between platelet level and BRVO formation is not clear. It has been proposed in the previous study demonstrating no statistically significant difference between groups in regard to mean platelet volume (MPV). Possible explanation for such differential results was the patient selection bias. Different stages of BRVO may also correlate with platelet values.

There are several limitations in this study. First, it is a cross-sectional study design and relatively small cohort of patients included, which might have influence on the statistical significance. Second, other significant prognostic inflammatory cytokines were not recorded and taken into consideration as it would have an effect on BRVO. Third, we included all the ischemic and nonischemic BRVO patients; hence, there was a lack of sufficient data about relevance between NLR, PLR, and the subgroup of BRVO.

In conclusion, the present study revealed that patients with BRVO have higher NLR and PLR than the control group. According to those outcomes, NLR and PLR may be considered as a novel and inexpensive predictive tool for assessing the risk for BRVO. Further studies with larger sample size are needed to confirm the diagnostic utility of NLR and PLR on BRVO patients.

## Figures and Tables

**Table 1:** Baseline characteristics of study participants.

Characteristic	Control (*n* = 81)	BRVO (*n* = 81)	*p* value
Age (yrs)	59.83 ± 1.154	61.35 ± 1.177	0.29
Gender (M/F)	51/30	47/34	0.52
Hypertension	56 (25)	58 (23)	0.73
BCVA (log MAR)	0.29 ± 0.10	0.52 ± 0.20	<0.001
IOP	16.44 ± 0.38	16.05 ± 0.33	0.43

**Table 2:** Laboratory findings of study participants.

Characteristic	Control (*n* = 81)	BRVO (*n* = 81)	*p* value
Hemoglobin (g/dL)	12.5 ± 1.45	12.0 ± 1.65	0.08
Mean platelet volume (fl)	10.3 ± 1.00	10.6 ± 0.88	0.27
Platelet count, ×109	239.4 ± 30.76	248.7 ± 32.37	0.09
White blood cells, ×109	7.44 ± 1.32	7.20 ± 1.08	0.19
Neutrophils, ×109	2.94 ± 0.65	4.31 ± 1.40	<0.001
Lymphocytes, ×109	2.41 ± 0.54	1.88 ± 0.49	<0.001
NLR	1.30 ± 0.47	2.52 ± 1.18	<0.001
PLR	105.9 ± 34.86	142.5 ± 45.19	<0.001

## Data Availability

The data used to support the findings of this study are included within the article.
